# Capitalizing glycomic changes for improved biomarker-based cancer diagnostics

**DOI:** 10.37349/etat.2023.00140

**Published:** 2023-06-28

**Authors:** Maria Luísa S. Silva

**Affiliations:** University of Toronto, Canada; Unidade de Aprendizagem ao Longo da Vida, Universidade Aberta, 1269-001 Lisboa, Portugal

**Keywords:** Cancer, biomarkers, aberrant glycosylation, assays, sensitivity, specificity

## Abstract

Cancer serum biomarkers are valuable or even indispensable for cancer diagnostics and/or monitoring and, currently, many cancer serum markers are routinely used in the clinic. Most of those markers are glycoproteins, carrying cancer-specific glycan structures that can provide extra-information for cancer monitoring. Nonetheless, in the majority of cases, this differential feature is not exploited and the corresponding analytical assays detect only the protein amount, disregarding the analysis of the aberrant glycoform. Two exceptions to this trend are the biomarkers α-fetoprotein (AFP) and cancer antigen 19-9 (CA19-9), which are clinically monitored for their cancer-related glycan changes, and only the AFP assay includes quantification of both the protein amount and the altered glycoform. This narrative review demonstrates, through several examples, the advantages of the combined quantification of protein cancer biomarkers and the respective glycoform analysis, which enable to yield the maximum information and overcome the weaknesses of each individual analysis. This strategy allows to achieve higher sensitivity and specificity in the detection of cancer, enhancing the diagnostic power of biomarker-based cancer detection tests.

## Introduction

Glycosylation of newly synthesized proteins and lipids is an essential process that occurs within cells, which enables to modify the intrinsic characteristics of the precursor molecules and to modulate diverse biological events [1]. In the case of proteins, and among other roles, glycosylation (1) allows to change protein polarity and solubility due to the hydrophilic nature of the glycan, (2) controls the tertiary structure of proteins by influencing their folding, (3) protects proteins from the action of proteolytic enzymes, (4) provides information about protein localization in the cell, (5) mediates events of protein recognition by other proteins and by the immune system, allowing it to distinguish between proteins from normal and from altered or non-self cells, and (6) many glycans act as receptors for viruses, bacteria, and parasites [2–4].

Protein glycosylation can be of two types, namely *O*- and *N*-glycosylation, depending on the way the glycan is covalently attached to the protein backbone, and includes diverse pathways, in which several glycosyltransferases and glycosidases take part [2, 4, 5]. The presence of specific glycosylation enzymes in cells of different tissues or organs results in the production of diverse glycoproteomes, which can distinguish the location where glycoproteins were synthesized [6, 7]. Furthermore, in several pathological processes, alterations in glycan synthesis and in glycosylation pathways lead to the production of aberrant glycoproteins, whose presence can be indicative of a disease state. In fact, protein glycosylation changes that occur in a cell during the transformation into a disease state tend to produce more marked effects than alterations in protein expression, which can be explained by two reasons. First, glycans are metabolic products whose levels are directly dependent on the expression of the respective glycosyltransferases. Therefore, up- or down-regulation of the corresponding enzyme results in an amplified effect. Second, changes in protein glycosylation pathways have the potential to disturb all glycoproteins produced in the affected cell [8]. Aberrant glycosylation of proteins has been observed in diabetes, Alzheimer’s disease, and several autoimmune diseases including rheumatoid arthritis and systemic lupus erythematosus, cardiovascular, respiratory, renal, and hepatic diseases, and cancer [2, 9–15]. It has also been associated with stress and chronic inflammatory states [2, 16, 17].

In cancer development, a high number of alterations in protein glycosylation has been reported, which are attributed to (1) the altered expression of glycosyltransferases, (2) changes in the tertiary conformation of the protein backbone and of the glycan chain, (3) the variability of acceptor substrates, (4) the availability and abundance of the sugar nucleotide donors and cofactors and/or (5) the localization of the pertinent glycosyltransferases in the Golgi apparatus [15]. Changes in protein glycosylation comprise (1) the increase in sialylation (especially in α2,6- and α2,3-linked sialylation) and polysialic acid synthesis, (2) the increase in fucosylation, (3) the increase in *O*- and *N*-linked glycan branching (such as the synthesis of complex β1,6-branched *N*-linked glycans), (4) the shortening of *O*-glycans (with the synthesis of the disaccharide Thomsen-Friedenreich antigen [T antigen] and the monosaccharide *N*-acetylgalactosamine [GalNAc; Thomsen nouveau antigen (Tn antigen)], as well as their sialylated forms sialyl-T antigen [ST antigen] and sialyl-Tn antigen [STn antigen], respectively), (5) alterations in the synthesis of histo-blood group-related antigens [loss of A and B antigens and changes in the Lewis family antigens, namely an increased expression or neoexpression of sialyl Lewis a antigen (SLe^a^), SLe^x^ and SLe^y^], and (6) the increase in *Helix pomatia* agglutinin (HPA)-binding glycans [15, 18–22].

When the aberrant glycoforms are exclusively produced by cancer cells, they can be used as cancer biomarkers, providing thus the required specificity for the monitorization of the disease. Their detection in blood or other biological specimens, including biopsy samples, has the potential to be useful for the diagnosis, prognosis, and/or therapy management, according to the sensitivity and selectivity of the glycobiomarkers.

Currently, several serological biomarkers that are used in the clinical practice for cancer diagnosis and/or monitoring are glycoproteins (Table 1). Though all these glycoproteins have shown to present abnormal glycosylation in cancer, this differential feature is not exploited, and the majority of the corresponding analytical assays only detect the total protein level (through an immunoassay) and not the aberrant glycoform. Solely α-fetoprotein (AFP) and cancer antigen 19-9 (CA19-9) are clinically monitored for their glycan changes, and only the AFP assay includes quantification of both protein level and altered glycoform. On the other hand, the aberrant and cancer-associated glycoforms have shown, so far, limited applicability in cancer screening and diagnosis, as a result of their relatively low specificity [15].

**Table 1:** Cancer glycobiomarkers approved for clinical practice, for which aberrant glycoforms have already been reported (these two criteria—approval by official entities and presenting aberrant glycoforms associated with cancer) were used to select the markers to be included in the table)

**Serological glycobiomarker**	**Cancer type(s)**	**Altered glycosylation**	**Type of detection**	**Clinical applications**	**References**
AFP-L3	Germ-cell hepatoma non-seminomatous Testicular carcinoma	Core fucosylation of AFP-L3	AFP concentration and AFP-L3 glycoform concentration	Diagnosis Prognosis Staging Recurrence detection Therapy monitoring	[23–34]
CA15-3 (MUC1) CA27.29 (MUC1)	Breast carcinoma	Increased level of mannosylated *N*-glycans, sialylated *O*-glycans, truncated *O*-glycans (T, Tn, and respective sialylated derivatives)	MUC1 concentration (both assays use different antibodies that recognize diverse epitopes of the protein)	Therapy monitoring	[23–25, 35–37]
CA19-9	Pancreatic carcinoma Colorectal carcinoma Other gastrointestinal cancers, in combination with other biomarkers	Increased expression of SLe^a^	SLe^a^ concentration	Therapy monitoring Recurrence detection Tumour burden assessment	[25, 38–44]
CA125 (MUC16)	Ovarian carcinoma	Increased expression of truncated *O*-glycans (Tn, STn, T, ST, and core 2), Le^x^ antigen expression, increased expression of bi-antennary complex-type and high mannose-type *N*-glycans	MUC16 concentration	Prognosis Recurrence detection Therapy monitoring	[23–25, 45–51]
CEA	Colorectal carcinoma Gastric carcinoma Pancreatic carcinoma Breast carcinoma Lung carcinoma	Increased fucosylation and sialylation, increased expression of mannose, Le^x^, SLe^x^, Le^y^, and T antigen, increased branched *N*-glycans	CEA concentration	Staging Prognosis Recurrence detection Therapy monitoring	[23–25, 37, 52–57]
Total PSA Pro2PSA and free PSA	Prostate carcinoma	Altered fucosylation and sialylation, H2 epitope, increase in GalNAc content, LacdiNAc structure, α2,3-sialic acid on the terminal galactose of *N*-linked oligosaccharides	Total PSA and free PSA concentration	Screening Diagnosis (with digital rectal examination) Pro2PSA and free PSA are used to discriminate cancer from benign disease	[23–25, 58–61]
Tg	Thyroid carcinoma	Increased LCA-reactive Tg, altered sialylation and fucosylation, β1,6-branching of glycans, altered content and structure of poly-LacNAc chains, *O*-GlcNAcylation	Tg concentration	Therapy monitoring	[23–25, 62–69]

MUC1: mucin 1; CEA: carcinoembryonic antigen; LCA: *Lens culinaris* agglutinin; Le^x^: Lewis x antigen; PSA: prostate-specific antigen; LacdiNAc: GalNAcβ1,4-GlcNAc; Tg: thyroglobulin; LacNAc: *N*-acetyllactosamine; *O*-GlcNAcylation: *O*-linked-*N*-acetylglucosaminylation

This review aims to present several examples where the combined quantification of protein cancer biomarkers and the respective glycoform analysis have been performed, and which enable to clearly demonstrate the advantages of this approach *versus* the sole quantification of the protein concentration.

## Improved cancer diagnosis and monitoring based on the use of specific serum glycobiomarkers

### AFP and AFP-L3

AFP is an oncofoetal glycoprotein, largely expressed in foetal yolk sac and liver during embryonic development. Its concentration gradually decreases after birth to < 10 ng/mL in 12–18 months. During pregnancy, AFP levels rise again in maternal serum. AFP levels may also increase in several pathological situations, such as hepatocellular carcinoma (HCC), as well as in other gastrointestinal, lung, and testicular carcinomas [70]. Furthermore, it has also been reported to increase in benign liver diseases, namely in acute and chronic viral hepatitis and cirrhosis [70–72].

Although it is not specifically produced in HCC, AFP has been historically used as a tumour marker for this disease [26, 73]. Nevertheless, AFP expression may vary significantly in HCC, in a range from normal to > 100,000 ng/mL [74]. Up to 30% of HCC patients show normal AFP values at the time of diagnosis, remaining low even in advanced disease [75], and independently of the tumour size at diagnosis [76]. This results in large numbers of false negatives and low sensitivity of the test for HCC diagnosis [76, 77]. On the other hand, elevated AFP levels may be found in liver benign diseases, as mentioned before, resulting in high numbers of false positives and low specificity [26, 76]. Together, these findings rule out the use of AFP for HCC diagnosis and for screening purposes [76].

To improve the sensitivity and the diagnostic power of AFP for HCC, its glycoforms have been investigated using lectin electrophoretic techniques. It was found that AFP presents a distinctive affinity for LCA and, accordingly, three glycoforms can be separated, namely AFP-L1, AFP-L2, and AFP-L3. AFP-L1 does not bind to LCA and is the major glycoform of AFP in the serum of chronic hepatitis and cirrhosis. AFP-L2 is mostly resultant from yolk sac tumours and is also produced during pregnancy, showing an intermediate affinity for LCA [78]. AFP-L3 is the LCA-bound fraction of AFP, being produced in HCC even at early stages [26, 29].

**Figure 1:**
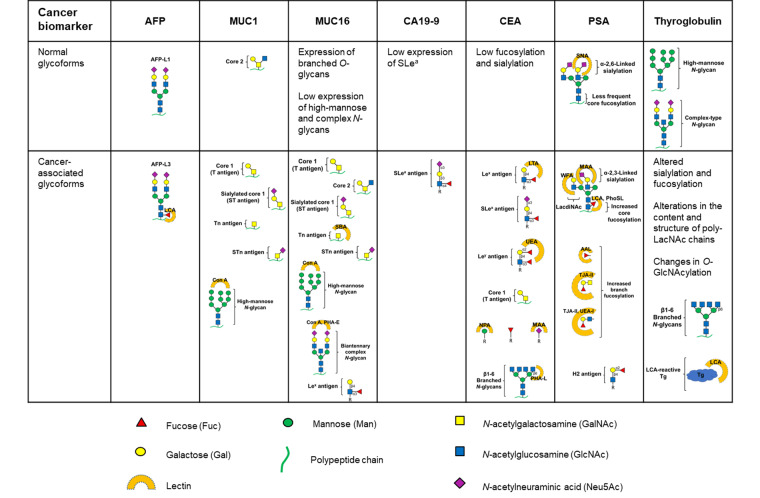
Glycan structures present in normal and cancer-associated glycoforms of the glycoproteins that are used in the clinic as cancer biomarkers. Lectins used for the selective binding of specific glycoforms are indicated next to the respective glycoform. Con A: concanavalin A; SBA: soybean agglutinin; PHA-E: *Phaseolus vulgaris* erythroagglutinin; LTA: *Lotus tetragonolobus* agglutinin; UEA: *Ulex europaeus* agglutinin; NPA: *Narcissus pseudonarcissus* agglutinin; MAA: *Maackia amurensis* agglutinin; PHA-L: *Phaseolus vulgaris* leucoagglutinin; SNA: *Sambucus nigra* agglutinin; WFA: *Wisteria floribunda* agglutinin; PhoSL: *Pholiota squarrosa* lectin; AAL: *Aleuria aurantia* lectin; TJA: *Trichosanthes japonica* agglutinin

AFP-L3 presents α1,6 core fucosylation on the reducing terminus of *N*-acetylglucosamine of AFP molecule, and this altered glycosylation provides the reactivity towards LCA (Figure 1). Elevated AFP-L3 levels in HCC result from overexpression of fucosyltransferase 8 (Fut 8; responsible for core fucosylation of proteins in the liver) [79] and also from the increased release of AFP-L3 from hepatocytes into plasma in HCC [80].

AFP-L3 has shown to be a highly specific biomarker for HCC, allowing to differentiate HCC from cirrhosis, and also serves as a predictive marker for the development of HCC during the follow-up of patients with cirrhosis [28, 30, 31]. The AFP-L3 percentage of the total AFP (AFP-L3%), calculated as [(AFP-L3/total AFP) × 100], has been used as a marker for early diagnosis of HCC, for therapy evaluation and to predict the prognosis of HCC [26, 32, 33], being particularly useful in the “diagnostic dilemma” of cases with total AFP concentrations between 10 ng/mL and 200 ng/mL and to exclude HCC in benign conditions with elevated AFP [27, 34]. The clinical performance of the AFP-L3% biomarker *versus* total AFP for the diagnosis and monitoring of HCC is presented in Table 2.

**Table 2:** Clinical performance of AFP-L3% *versus* AFP as serological biomarkers for HCC (representative examples published since 2007)

**Assay**	**Sensitivity (%)**	**Specificity (%)**	**Other analytical features and advantages**	**Limitations**	**Reference**
AFP total concentration quantification					
Multiple reaction monitoring—MS	76.0 (optimum cut-off 6.00 ng/mL)	77.5 (optimum cut-off 6.00 ng/mL)	-	-	[81]
LiBA μTAS (Wako i-30 autoanalyzer)	59.0 (optimum cut-off 5.90 ng/mL)	89.5 (optimum cut-off 5.90 ng/mL)	Total AFP concentration range from 0.3 ng/mL to 4,000 ng/mL	-	[81]
LiBA μTAS (Wako i-30 autoanalyzer)	33.6 (cut-off 200 ng/mL)	98.0 (cut-off 200 ng/mL)	-	-	[82]
AFP-L3% quantification					
LiBA μTAS (Wako i-30 autoanalyzer)	72.4 (cut-off 7.7%)	90.9 (cut-off 7.7%)	Sensitivity and specificity remained practically constant, independently of the AFP total concentration	-	[83]
Liquid chromatography—parallel reaction monitoring—MS	80.8 (cut-off 18.6%)	100 (cut-off 18.6%)	Early detection of HCC with higher accuracy than μTAS Analytical performance identical to μTAS conventional system with better sensitivity and accuracy	Sample preparation prior to MS analysis is required More time-consuming than microarray systems	[83]
Multiple reaction monitoring—MS	81.0 (optimum cut-off 0.132%)	89.5 (optimum cut-off 0.132%)	Early detection of HCC (screening) Lower incidence of false negatives compared to LiBA assay AFP-L3 concentration range from 0.132% to 100%, with a LLOQ of 0.051 ng/mL AFP	-	[81]
LiBA μTAS (Wako i-30 autoanalyzer)	61.5 (optimum cut-off 0.500%)	90.0 (optimum cut-off 0.500%)	AFP-L3 concentration range from 0.5% to 99.5%, with a LLOQ of 0.3 ng/mL AFP	AFP-L3% cannot be reported if the total AFP concentration is < 0.3 ng/mL, even in cases of high AFP-L3 concentrations	[81]
LiBA μTAS (Wako i-30 autoanalyzer)	41.5 (cut-off 5% and total AFP concentration < 20 ng/mL) 36.2 (cut-off 5% and total AFP concentration < 10 ng/mL)	85.1 (cut-off 5% and total AFP concentration < 20 ng/mL) 88.5 (cut-off 5% and total AFP concentration < 10 ng/mL)	Better diagnostic performance than the conventional LiBA system assay for low serum total AFP concentrations (< 20 ng/mL) AFP-L3% correlated with poor prognosis and low survival rate LLOQ of 0.3 ng/mL AFP	-	[84]
LiBA μTAS (Wako i-30 autoanalyzer)	60.0 (optimum cut-off 7% and total AFP concentration 200 ng/mL) 41.1 (optimum cut-off 7% and total AFP concentration < 20 ng/mL)	90.3 (optimum cut-off 7% and total AFP concentration 200 ng/mL) 91.9 (optimum cut-off 7% and total AFP concentration < 20 ng/mL)	More sensitive in discriminating HCC than the corresponding LiBA system and total AFP concentration assays, even for low total AFP concentrations Has predictive value for long-time survival Short-time and automated assay	-	[82]
LiBA (Wako LiBASys clinical autoanalyzer)	71 (cut-off ≥ 10%) 33 (cut-off ≥ 35%)	63 (cut-off ≥ 10%) 100 (cut-off ≥ 35%)	Total AFP concentration range from 10 ng/mL to 200 ng/mL, with a LLOQ of 0.8 ng/mL AFP and 0.5% AFP-L3 AFP-L3%, in combination with the AFP level, increases the specificity of diagnosis of HCC in individuals with indeterminate increases of total AFP level (10–200 ng/mL)	-	[27]

-: blank cell; LLOQ: lower limit of quantification; MS: mass spectrometry; LiBA: liquid-phase binding assay; μTAS: micro-total assay system

Comparing the results obtained for AFP-L3% and total AFP quantification, when performed on the same samples, it is clear that AFP-L3% has superior behaviour, with improved sensitivity and specificity, demonstrating the benefits of quantifying this glycobiomarker instead of only measuring the total protein concentration.

Recent studies on fucosylated glycoforms of other liver-secreted proteins, namely haptoglobin, kininogen, α1-antitrypsin and Golgi protein 73 have shown the promising potential of these glycobiomarkers for differential diagnosis of HCC *versus* cirrhosis with improved sensitivity and specificity [85, 86]. In addition, the monosialylated AFP (msAFP) has also proved to be more specific than AFP, showing the potential to be a diagnostic marker for HCC in nondiagnostic AFP conditions [87].

### MUC1 and its cancer-associated glycoforms

MUC1 is a heavily glycosylated transmembrane protein (with 50% to 90% of its molecular mass corresponding to carbohydrates), that belongs to the MUC family. It is physiologically expressed in breast tissue, specifically on the glandular or luminal epithelial cells of the mammary gland, as well as in the apical surfaces of simple secretory epithelia of gastrointestinal, respiratory, urinary, and reproductive tracts [88], protecting the underlying epithelia.

In most human epithelial cancers, MUC1 is overexpressed and exhibits aberrant glycosylation [89–92]. In breast cancer cells there is increased sialylation of MUC1, which results in truncation of sugar branches that would be otherwise elongated. Therefore, MUC1 presents mainly core 1 *O*-glycans (namely T antigen) in opposition to the extensively branched core 2 *O*-glycans carried by normal MUC1, and the Tn antigen is also overexpressed, as well as the respective sialylated derivatives (ST and STn antigens) [91, 93–95]. There is also an increased expression of high-mannose-type *N*-glycans [96]. In cancers located in other tissues, different aberrant glycosylation of MUC1 occurs [55, 92, 97].

The CA15-3 assay detects the soluble forms of MUC1 (the peptide backbone), and it is used for therapy monitoring in patients with metastatic disease [98–100]. Since the referred forms are also secreted from cells in other tumours and diseases, the assay lacks the required specificity for breast cancer. Besides, the assay also presents low sensitivity for *in situ* or low stage disease, as the levels of the soluble forms of MUC1 rarely increase at the early stages of breast cancer. Therefore, it cannot be used for screening or early detection purposes [91, 98]. A related assay, known as CA27.29, also detects the soluble forms of MUC1 but uses a different antibody and a different detection strategy. Both assays originate similar but not identical results [101]. Although it seems that the CA27.29 assay has greater sensitivity than the CA15-3 assay [102], its routine use is yet not recommended due to insufficient data [100, 103].

Aberrant glycoforms of MUC1 have been evaluated in order to be used as breast cancer biomarkers with improved clinical performance over the conventional CA15-3 and CA27.29 assays. An antibody-lectin sandwich assay was designed to detect the aberrant glycosylation of MUC1 in sera from breast cancer patients, using an antibody to capture the protein and the lectin Con A to detect high mannose *N*-glycans present in MUC1 [35]. The results revealed that mannosylated *N*-glycans of cancer-associated MUC1 were the most effective targets for the antibody-lectin sandwich assay. In addition, it was shown that the glycosylation level increased with the progression of the disease and the assay efficiently discriminated breast cancer stage I (sensitivity: 63%, specificity: 69%), IIA (sensitivity: 77%, specificity: 75%), IIB (sensitivity: 69%, specificity: 86%) and III (sensitivity: 80%, specificity: 65%) from benign breast disease. There were no differences in the mannosylated *N*-glycan level of MUC1 between the normal and the benign groups. Immunodetection of the MUC1 protein revealed no differences between the groups in a sandwich enzyme-linked immunosorbent assay (ELISA) using two different anti-MUC1 antibodies, confirming that the increased response of cancer samples in the developed assay was due to the increased *N*-glycosylation of MUC1 rather than to an increased concentration of MUC1. The enhanced sensitivity of the developed assay *versus* the current ELISA CA15-3 assay was justified by the heavy glycosylation of MUC1 that resulted in an amplified analytical signal. Although a larger sample size is needed to validate the reported findings, the study demonstrated the advantages of detecting the aberrant glycosylation of the biomarker instead of the total protein concentration.

### MUC16 and its cancer-associated glycoforms

MUC16 is a large membrane glycoprotein that belongs to the MUC family. It is highly *O*-glycosylated, with a carbohydrate content accounting for approximately 77% of the total weight of the MUC [104]. CA125 is an epitope present on MUC16, which is detected by specific monoclonal antibodies, such as OC125 and M11, used in CA125 immunoassays [105]. MUC16 is physiologically expressed by secretory epithelial cells of the eye, oral cavity, heart, lung, gastric tract, gall bladder, cervix, and uterus, providing lubrification and protection to the epithelial surfaces [106]. In healthy populations, CA125 serum levels are < 35 U/mL [107], and the values tend to decline with aging and menopause. On the other hand, elevated CA125 levels can be found in healthy women, during the follicular phase of the menstrual cycle [108] and during the first trimester of pregnancy [109], and in non-malignant diseases such as cirrhosis, hepatitis, endometriosis, and ovarian cysts [110–112].

During cancer development, MUC16 is aberrantly expressed, both in its amount and glycosylation [46, 113, 114]. Aberrant MUC16 has been reported to be produced in diverse carcinomas such as ovarian, lung, breast, gastrointestinal, kidney, cervical, uterine, and endometrial [48, 49]. Cancer-associated CA125 antigen presents both *N*- and *O*-glycosylation, with *N*-linked glycans being mainly composed of bi-antennary complex-type and high mannose-type oligosaccharide chains, and *O*-glycans are, predominantly, mono- and disialylated core type 1 (Galβ1,3-GalNAc) and core type 2 glycans [Galβ1,3-(GlcNAcβ1,6)-GalNAc]. The majority of *O*-glycans are sialylated and/or fucosylated, and carry a maximum of two fucoses or two sialic acids, or two fucoses and two sialic acids. The Le^x^ epitope is also present, both in *O*- and *N*-glycans [47].

Since MUC16 can be overexpressed in a variety of non-ovarian cancers and benign diseases, the CA125 assay shows poor specificity for ovarian cancer diagnosis and frequently presents false positives. Additionally, the assay has also low sensitivity for ovarian cancer, as around 50% of patients with early stages of ovarian cancer do not express elevated CA125 values, resulting in false negatives [115]. Therefore, the CA125 assay is not recommended for screening ovarian cancer in asymptomatic women. However, it is recommended for screening women with a family history of ovarian cancer, in conjunction with other imaging tests. Furthermore, CA125 testing is recommended as an adjunct to discriminate between benign and malignant suspicious pelvic masses, particularly in postmenopausal women. In spite of its limited sensitivity and specificity for ovarian cancer, CA125 concentration correlates with disease stage, tumour size, and prognosis, so its measurement is recommended and useful for monitoring the response to therapeutic treatment and for evaluation of disease status and detection of recurrence. As CA125 concentration may vary due to differences in calibration, assay design, and reagent specificities, it is recommended that the same assay method should be used throughout for a single patient [99].

Other serum biomarkers have been proposed for ovarian cancer detection, namely human epididymis protein 4 (HE4), alone or in combination with CA125, but the available data are insufficient to conclude a significant improvement in the clinical performance of the assays *versus* the CA125 assay alone [116]. Multiple-marker based algorithms, namely ROMA and OVA1, have also been developed and approved to assess ovarian cancer risk in women with a pelvic mass, showing increased benefit over the traditional CA125 assay. However, they are not recommended for ovarian cancer screening or diagnosis and the results must be interpreted together with an independent clinical and radiological evaluation [117, 118].

The glycosylation profile of CA125 antigen from human amniotic fluid and from a human ovarian carcinoma cell line 3 (OVCAR-3) was compared, using lectin-affinity chromatography [46]. The results showed a higher abundance of *O*-glycans in the CA125 antigen from the ovarian cancer cells, with higher expression of GalNAc and lower expression of Galβ1,3-GalNAc, compared to the amniotic fluid samples. Also, the terminal GalNAcβ1,4-GlcNAc structure was found to be predominantly present in ovarian cancer cells. High-mannose-type and bisecting GlcNAc *N*-glycans were also found to be more abundant in ovarian cancer cells. Although this was a preliminary study, it demonstrated the differential glycosylation pattern between CA125 antigens from normal and cancer origins, and the potential use of a lectin-based assay to discriminate CA125 glycoforms and to complement the total CA125 concentration test, improving its diagnostic performance towards the detection of ovarian cancer. In another study, a sandwich ELISA was developed to measure the level of STn antigen expressed on MUC16, which allowed to differentiate between patients with endometriosis and ovarian cancer [50]. An anti-MUC16 monoclonal antibody was used for capture and an anti-STn monoclonal antibody was used for detection. It was shown that STn antigen was noticeably detectable in the MUC16-enriched fractions from OVCAR-3 cells but negligible in those from peritoneal fluid of patients with endometriosis, and that the level of STn-MUC16 was significantly higher in the samples from patients with ovarian cancer, compared with the samples from patients with endometriosis. These results were consistent with immunohistochemical analysis. Furthermore, the levels of MUC16 and STn-MUC16 were compared for different clinical stages and cytological grades of ovarian cancer, and it was found that an elevated level of STn-MUC16 was more closely associated with an advanced clinical stage and cytological grade than that of MUC16 concentration alone. The level of STn-MUC16 discriminated patients with endometriosis and with ovarian cancer, with 44% sensitivity and 100% specificity.

A microarray-based platform has also been developed for profiling specific aberrant glycoforms including T, Tn, and STn antigens, present on CA125 (MUC16) and CA15-3 (MUC1) [51]. A blinded cohort study of patients with elevated CA125 levels (30–500 kU/L) and a pelvic mass, from the UK Ovarian Cancer Population Study (UKOPS), was performed by analysing a combined glycoform profile that comprised STn-CA125, ST-CA125, and STn-CA15-3 (the three best-performing glycoforms). Glycoprofiling analysis was able to distinguish benign ovarian neoplasms from invasive epithelial ovarian/tubule cancer with a specificity of 61.1% at 90% sensitivity, whereas the CA125 specificity for the commercial assay was only 41.3% at the same sensitivity. The developed microarray assay thus showed its potential to improve the differential diagnosis of ovarian cancer and to significantly reduce the number of patients elected for further testing. A refinement of this study was achieved through the use of proximity-ligation assay (PLA), in which aberrant glycoforms (Tn, STn, and T) of MUC16 and MUC1 in ovarian cancer tissue were detected [119]. PLA reactions for Tn-MUC16, STn-MUC16, Tn-MUC1, and STn-MUC1 were negative in benign lesions but often positive in borderline and malignant lesions. The developed assay allowed to improve the analytical performance of the previous microarray assay, increasing the sensitivity to 72% and 83% in two series of samples, with 100% specificity. The described examples reinforce the usefulness of employing the analysis of MUC16 aberrant glycoforms to improve the clinical performance of MUC16 as a biomarker for ovarian cancer.

### CA19-9

The CA19-9 antibody recognizes a glycan epitope present in MUC-type glycoproteins in serum, and in glycolipids (monosialogangliosides) in tissues. The immunodeterminant structure is SLe^a^ [Siaα2,3-Galβ1,3-(Fucα1,4)-GlcNAc], a sialylated glycoform of the Le^a^ blood group antigen [120, 121]. The CA19-9 antibody reacts with some normal tissues, namely the pancreatic, gall bladder, and gastric epithelia, showing that CA19-9 can be expressed under physiological conditions [120]. Nevertheless, CA19-9 is also expressed in cancer, for example, carried by MUCs which are known to play a role in cancer progression and metastization [121].

CA19-9 has been extensively reported to be a useful diagnostic aid for pancreatic cancer and it is the gold standard biomarker for this disease, being used to discriminate malignant from benign disorders of the pancreas [42, 43]. Elevated levels of CA19-9 in pancreatic cancer patients have also been associated with disease recurrence [44] and poor prognosis [39]. CA19-9 can also be elevated in other gastrointestinal, ovarian, and hepatocellular cancers, although its routine measurement in such cases is not recommended [40, 99, 121]. However, elevated CA19-9 levels can also be found in benign diseases (and even in healthy individuals, depending on their genetic variation for the expression of CA19-9 and related epitopes) [122], and in about 20% of the patients with pancreatic adenocarcinoma its level may be normal [40, 121].

Due to its insufficient specificity and sensitivity, CA19-9 is not recommended for screening, diagnosis, staging, surveillance, or treatment monitoring of patients with pancreatic or colorectal cancer (CRC) [40, 103]. However, it can be used to monitor the response to therapy for locally advanced metastatic pancreatic cancer and the increase in CA19-9 levels may indicate disease progression [40].

CA19-9 is a carbohydrate and, thus, the aberrant glycosylation associated with cancer is already capitalized in this biomarker. Still, the clinical performance of CA19-9 in the early diagnosis of pancreatic cancer can be further extended if other cancer-associated aberrant glycans and proteins are detected in combination with CA19-9. In fact, a wide range of glycosylation alterations have been associated with pancreatic cancer such as increased expression of SLe^x^ antigen, increased expression of truncated *O*‑glycans (Tn and STn), increased branched and fucosylated *N*‑glycans, upregulation of specific proteoglycans and galectins, and increased *O*‑GlcNAcylation (Figure 1) [123]. For example, it was shown that CA19-9 expression in MUC5AC and MUC1 proteins may be increased, independently of protein elevation, and that the combined measurement of MUC5AC and CA19-9 presents better performance and improved specificity to differentiate pancreatic cancer subjects from controls [124, 125]. At a threshold of 96% specificity, 78% and 83% of pancreatic cancer patients showed increased expression of CA19-9 in MUC5AC and MUC1, respectively [124]. The complementary relationship between protein and glycan elevations resulted in better sensitivities achieved when both glycan and protein were measured, *versus* measuring just the protein. In comparison, the median sensitivity of CA19-9 for the diagnosis of pancreatic cancer calculated from pooled data is 79% (70–90%) with a median specificity of 82% (68–91%) [43]. Furthermore, the combined detection of CA19-9 and other cancer-associated aberrant glycostructures, namely wheat germ agglutinin (WGA)-binding glycans in serum MUCs [126], CA242 [127], CA72-4 [128, 129] or SLe^x^ [130, 131] enabled to increase the sensitivity and specificity of the assays, compared to the measurement of CA19-9 alone. The advantageous combination of CA19-9 with other cancer-associated proteins or glycostructures has also been demonstrated for the detection of gastric cancer [132–134].

### CEA and its cancer-associated glycosylation

CEA is a highly glycosylated (with approximately 60% carbohydrate content) complex glycoprotein belonging to the immunoglobulin gene superfamily [135]. It is an oncofoetal antigen, produced by the intestinal tissue of a normal foetus, but expressed in normal adults only in trace amounts. Being a cell surface immunoglobulin, CEA functions are related with cell adhesion, intercellular recognition, and attachment, exerting also immunoregulatory roles [52, 136]. CEA has been detected in the glycocalyx of surface epithelial cells of normal colon mucosa [137], and more than 95% of the normal population expresses CEA in levels below 5 ng/mL. Smoking habits somewhat increase CEA normal serum values, and CEA tends also to slightly increase in older people [138, 139]. CEA can also be increased in non-malignant diseases, such as liver, gastrointestinal, renal, and other chronic diseases [138, 140, 141].

CEA is also expressed in diverse carcinomas including breast [142], gastric [143], pancreatic [144], renal [145], and lung [146]. In colon carcinogenesis, CEA overexpression contributes to disturb the normal histological architecture of colonic epithelium [147] and facilitates cellular migration and metastization [148].

Both in benign and malignant diseases there is no progressive increase in CEA levels, with values higher than 10 ng/mL being rarely observed [138]. In addition, although CEA is expressed in the great majority of CRCs, its levels are usually normal at the time of diagnosis due to the first-pass effect in CEA metabolization [149]. In spite of its low specificity and sensitivity for CRC diagnosis, CEA has been extensively used as the main biomarker for CRC, as well as for tumours of the gastrointestinal tract. Although it is not recommended for CRC screening, it can be used for staging (as increased serum levels correlate with advanced stages) and for surgical treatment planning. Elevated preoperative CEA may correlate with poorer prognosis and it is recommended that postoperative serum CEA testing may be performed every 2–3 months in patients with stage II or III disease or for ≥ 2 years after diagnosis, as an increase in postoperative CEA level may indicate recurrence. CEA is the marker of choice for monitoring metastatic CRC during systemic therapy [40, 52, 99, 150].

CEA exhibits extensive glycosylation on asparagine residues with up to 28 complex *N*-linked multi-antennary glycan chains with Le^x^ and SLe^x^ structural motifs [57], with the glycofraction consisting mainly of *N*-acetylglucosamine, mannose, galactose, fucose and sialic acid [150]. *O*-glycosylation is quantitatively less important than *N*-glycosylation [55]. The *N*-glycosylation profile of CEA purified from human colon carcinoma cells and from human liver metastases of CRC cells was analyzed by MS, showing distinctive features *versus* normal CEA *N*-glycosylation pattern, namely simultaneously increased bisection and branching, incomplete galactosylation or poly-LacNAc elongation on highly branched structures, moderate levels of sialylation and high levels of fucosylation (Figure 1) [53]. Overexpression of bi-antennary, tri-antennary, and tetra-antennary structures carrying sialic acid and fucose residues, as well as terminal SLe^x^ structure, were also reported in another study, where CEA from samples of human CRC tissue were analyzed (Figure 1) [54]. In this study, approximately 50% of CEA glycans presented core fucosylation. The glycosylation of CEA from normal *versus* tumour colon tissue was also characterized using a different approach, based on lectin binding and ELISA assays [55]. The results revealed that tumour-associated CEA contained high levels of Le^x^ and Le^y^ antigens (proved by the strong binding of LTA and UEA, respectively), as well as increased expression of mannose (shown by increased binding of NPA) and β1,6-branched *N*-glycans (demonstrated by strong binding of PHA-L). MAA binding was higher for the cancer samples, evidencing increased expression of sialic acid (Figure 1). The study demonstrated the feasibility of differential detection of tumour-associated CEA using lectin-based ELISA assays and a panel of lectins. A high-density lectin microarray comprising 56 lectins was also developed for the analysis of the glycosylation pattern of CEA from tumour tissues of CRC patients [56]. The results showed different CEA glycosylation profiles between normal and cancer tissues, with CEA from tumour tissues presenting the higher expression of fucose (both terminal and core), mannose and T antigen, and lower expression of GalNAc, *N*-acetylglucosamine, galactose, and branched and bisecting *N*-glycans. Moreover, a combinatorial assessment of nine lectins was found to be sufficient to distinguish CRC tumour tissues from tumour-adjacent normal tissues with 83% sensitivity and around 90% specificity. The reported clinical performance surpassed the average performance of the total CEA concentration assay (sensitivity of ~80% and specificity of ~70%) [151]. Additionally, the developed assay showed that alterations in the glycosylation pattern of CEA correlated well with CRC tumorigenesis and progression and that specific glycans were differently expressed on CEA in a stage-dependent manner. In spite of the scarce quantitative studies regarding the improved performance of CEA cancer-associated glycoforms over the conventional total CEA concentration assay, the above-referred studies corroborate the importance of glycosylation analysis of CEA to enhance the clinical performance of this CRC biomarker.

### PSA and its cancer-associated glycoforms

PSA is a glycoprotein primarily expressed in prostate tissue [152]. It is produced almost exclusively by the luminal epithelial cells of prostatic glandular tissue in benign and malignant situations, which results in its low specificity in discriminating both. Most frequently, elevated values of PSA are indicative of benign prostatic hyperplasia (BPH), being also elevated in cases of urinary retention, prostatitis, and ejaculation [153, 154].

Total PSA serum concentration remains the most commonly used cancer biomarker for early detection of prostate cancer, and to monitor therapeutic response, aggressiveness, and recurrence [155, 156]. Nonetheless, its use is limited since there is no single cut-off value that simultaneously yields the high sensitivity and specificity required for a screening test [157–159]. In fact, there is a continuum of risk associated with PSA level and no PSA cut-off point can be clearly defined at which biopsy is recommended [160]. Furthermore, total PSA concentration cannot discriminate between potentially aggressive and indolent prostate cancers [161]. Due to its poor specificity, total PSA quantification is often associated with the risk of overdiagnosis [155, 162] which leads to overtreatment and the associated side effects such as sexual dysfunction, urinary incontinence, bowel problems, together with increased healthcare costs [163].

Other molecular forms of PSA and their ratios, namely free PSA and percent free PSA, proenzyme PSA (proPSA), its derivatives, and the prostate health index, as well as PSA derived-parameters and kinetics (PSA-density, PSA-velocity and PSA-doubling time), have been developed and tested, aiming to improve the specificity of the assays for the diagnosis of prostate cancer and for the differentiation between indolent and aggressive prostate cancers. Although these novel biomarkers have shown better clinical performance than total PSA quantification, allowing to reduce unnecessary biopsies while maintaining high specificity for prostate cancer [164–167], they also present some technical and clinical limitations [168, 169].

The PSA glycosylation profile has been investigated in order to detect cancer-specific alterations that can be employed as efficient prostate cancer glycobiomarkers. Several differences in glycosylation have been found between PSA from normal and from tumour origins (Figure 1), namely in the outer ends of bi-antennary complex structures. PSA produced in cancer cells shows a higher fucose content (core fucose linked α1,6 to GlcNAc) and the oligosaccharides are neutral (do not contain sialic acid), in contrast to normal PSA glycans, which are sialylated. Fucose is also linked α1,2 to galactose, forming the H2 epitope, absent in PSA from normal cells. This is related with the expression of α1,2-fucosyltransferase in tumour cells. There is also an increase in GalNAc content in PSA from tumour cells (from 25% to 65%) [58]. Furthermore, a sialic acid-α2,3-galactose linkage was found as an additional terminal carbohydrate structure, which is detected by MAA [60]. The main glycosylation changes found in PSA from cancer origin and the advantages of detecting the aberrant glycoforms over the conventional detection of PSA protein concentration are presented in Table 3.

**Table 3:** Main glycosylation changes found in PSA from cancer origin and the advantages of the corresponding detection assays over the conventional PSA tests (representative examples published since 2007)

**Alteration in glycosylation (*versus* normal PSA)**	**Assay**	**Reported main advantages over PSA conventional detection**	**Limitations**	**Reference**
Increased total fucosylation	Solid-phase permethylation and MALDI-MS	Pre-screening method to aid early PC detection	**-**	[170]
Higher content of α2,3-linked sialic acid Lower content of α2,6-linked sialic acid	Lectin immunosorbent assay, using SNA, MAA-I, and MAA-II	Low limits of detection (0.04–1.35 ng/mL) and good reproducibility (%CVs < 10%) Total SNA assay (AUC = 0.71) performed better than %fPSA (AUC = 0.54) in its diagnostic grey zone between 10% and 20% Direct analysis of PSA glycosylation in sera High-throughput assay Sufficient limit of detection to analyze PSA sialylation in non-cancer sera with < 10 ng/mL of PSA	A larger sample size would be needed to validate the reported findings The assay should be completed with the inclusion of immunosorbent assays using lectins that recognize other carbohydrate moieties (e.g., fucose)	[171]
Increased expression of α1,2-linked fucose and β-GalNAc residues	Lectin-affinity chromatography and ELISA, using TJA-II	TJA-II-bound PSA content and TJA-II binding ratios (%) could be used to discriminate between PC and BPH with more than a 95% probability	**-**	[61]
Increased fucosylation of fPSA	Enzyme-linked immunosorbent lectin assay, using UEA-I	92% Specific and 69% sensitive for PC over BPH; in comparison, fPSA measurement was 70% specific and 56% sensitive (threshold set to 25% tPSA) for PC over BPH (PSA in the range of 4–10 ng/mL) Methodology compatible with equipment available in the majority of biomedical laboratories	A larger sample size would be needed to validate the reported findings	[172]
Increased levels of core-fucosylated bi-antennary glycans and α2,3-linked sialic acids Decreased tri-antennary tri-galactosylated glycans and tetra-antennary tetra-sialylated glycans with outer arm fucose	Serum *N*-glycome release followed by NP- and ccc with fluorescence detection	All chromatographic peaks significantly differentiated PC patients from BPH patients, with improved AUC values over PSA itself The glycan level may work in a more general patient population than the free/total PSA ratio Decreases in tri-antennary tri-galactosylated glycans and/or bisected core fucosylated biantennary monosialylated glycans and increases in tetra-antennary tetra-sialylated glycans correlated with tumour spread and patients’ survival	A larger sample size would be needed to validate the reported findings	[173]
GalNAcβ1−4GlcNAc-linked PSA (LacdiNAc-PSA)	Immunoassay system with SPFS, using anti-PSA IgG antibody to capture PSA and WFA for the detection of LacdiNAc	Limit of quantification of 0.256 pg/mL tPSA, with a dynamic range of at least five digits and limit of detection of LacdiNAc-PSA of 20.0 pg/mL AUC for LacdiNAc-PSA (0.851) was significantly greater than that for tPSA (0.559), and the optimum cut-off gave low false positivity (40.7%) and high sensitivity (88.4%), with specific distinction between PC and BPH within the PSA grey zone (4.0–20.0 ng/mL PSA) Comparing with recently reported PSA assays, LacdiNAc-PSA was significantly better than pro-PSA and fPSA Reduced assay time and minimum consumption of reagents	A larger sample size would be needed to validate the reported findings The detection could be further improved by reducing the influence of nonspecific reactions between fluorescently labelled WFA and serum proteins	[174]
α2,3-Linked sialylation as an additional terminal *N*-glycan on fPSA (S2,3PSA)	Magnetic microbead-based immunoassay, using anti-human fPSA monoclonal antibody (8A6) for capture and anti-α2,3-linked sialic acid monoclonal antibody (HYB4) for detection	AUC of 0.84, a sensitivity of 90.6%, and specificity of 64.2% for the diagnosis of PC with S2,3PSA (higher than those with PSA or %fPSA)	A larger sample size would be needed to validate the reported findings	[175]
Increased fucosylation of PSA	Lectin-affinity capturing of fucosylated glycoprotein (using AAL) and protein-antibody immunoreactivity	The fucosylated PSA achieved a better predictive power (AUC = 0.7056) when compared with tPSA (AUC = 0.6558) Using the ratio of fucosylated PSA as a predictive marker, it achieved even better performance when compared with the total serum PSA, in discriminating aggressive from non-aggressive tumours	A larger sample size would be needed to validate the reported findings	[176]
Decrease in core fucose Increase in α2,3-sialic acid percentage of PSA	Free PSA immunopurification followed by enzyme-linked lectin assay with PhoSL (detection of core fucosylation) and SNA lectin-affinity chromatography (detection of PSA α2,3-linked sialic acid level)	A cut-off value of 0.86 of the PSA core fucose ratio could distinguish high-risk PC patients from BPH with 90% sensitivity and 95% specificity, with an AUC of 0.94 A cut-off value of 30% of the α2,3-sialic acid percentage of PSA discriminated between high-risk PC and the groups of BPH, low-, and intermediate-risk PC, with a sensitivity of 85.7% and specificity of 95.5%, and an AUC of 0.97 (better than using tPSA or %fPSA in the diagnostic grey zone)	A larger sample size would be needed to validate the reported findings	[177]
Increased α2,3-linked sialyl *N*-glycan-carrying PSA ratio (%S2,3PSA)	Automated micro-total immunoassay system (μTAS system) using MAA	The limit of detection of S2,3PSA was 0.05 ng/mL with a %CV < 3.1% The AUC for the detection of PC for the S2,3PSA ratio (%S2,3PSA) with a cut-off value of 43.85% (0.8340) was much superior than the total PSA (AUC = 0.5062) The optimum cut-off point giving high specificity (72.0%) at 80% sensitivity was determined to be 42.20% of %S2,3PSA (significantly higher than that for conventional PSA testing) Assay time < 10 min	A larger sample size would be needed to validate the reported findings	[178]
Increased fucosylation of PSA	Lectin immunoassays using LCA and AAL followed by clinical PSA immunoassay	Fucosylated PSA-AAL had the best performance (AUC = 0.909), followed by total PSA (AUC = 0.881), fucosylated PSA-LCA (AUC = 0.839), %fucosylated PSA-AAL (AUC = 0.822), and %fucosylated PSA-LCA (AUC = 0.594) Fucosylated PSA-AAL, %fucosylated PSA-AAL, and fucosylated PSA-LCA levels could be effective biomarkers to differentiate aggressive PC from non-aggressive disease The combined use of fucosylated PSA and %fucosylated PSA could be used in place of tPSA with potentially improved performance in identifying aggressive disease	A larger sample size would be needed to validate the reported findings	[179]

AUC: area under the curve; CV: coefficient of variation; %fPSA: percentage of free PSA; MALDI-MS: matrix-assisted laser desorption/ionization MS; NP: normal phase; PC: prostate cancer; SPFS: surface plasmon field-enhanced fluorescence spectrometry; tPSA: total PSA; WAX: weak anion exchange; IgG: immunoglobulin G; HPLC: high-performance liquid chromatography

From Table 3, we can conclude that the detection of PSA glycoforms shows superior clinical performance and significant advantages over PSA tests, in terms of increased sensitivity and specificity, particularly when PSA concentration is in the diagnostic grey zone, being useful to distinguish between aggressive and indolent prostate cancers and to avoid unnecessary biopsies [180].

### Tg and its cancer-associated glycoforms

Tg is a glycoprotein (with around 10% of its molecular weight comprised of carbohydrates) produced by thyroid cells, being the most abundant glycoprotein in the thyroid gland. Tg plays an essential role in the synthesis of thyroid hormones since it is the initial block to which iodine is incorporated to form triiodothyronine (T3) and thyroxine (T4) [181]. Tg is predominantly *N*-glycosylated (although it also shows *O*-glycosylation), containing 20 putative *N*-glycosylation sites [182]. The main types of glycans in human Tg are high-mannose and bi-antennary complex-type structures [183, 184], and the majority of carbohydrate chains are sialylated [185]. *O*-glycans in Tg are still not fully identified [186]. The glycan components of Tg are important for the physiological functions of this glycoprotein, including Tg transport, hormone synthesis, and Tg antigenicity [187–189]. For example, sialylation was found to significantly affect the immunoreactivity and solubility of Tg, being also important for the binding to its transmembrane transporter [186, 190].

Measurement of Tg serum concentration is the routine procedure in the follow-up of patients with thyroid carcinoma, since elevated levels of serum Tg after total thyroidectomy indicate residual or recurrent disease [191]. It can also be used for the diagnosis of several thyroid benign conditions [186]. Serum Tg quantification is performed by immunometric methods, which include immunoradiometric and immunochemiluminometric assays [192, 193]. Although most recent methods are very sensitive, detecting Tg concentrations down to 0.1 ng/mL, they present several limitations [194]. First, as they are based on monoclonal anti-Tg antibodies, and assays from different providers use diverse antibodies that recognize different Tg epitopes, a significant variability in results is observed when diverse assays are used [193, 195]. Therefore, it is recommended that serum Tg should be assessed in the same laboratory and using the same assay during the follow-up of an individual patient [191]. In addition, monoclonal anti-Tg antibodies are produced from normal thyroid tissue-derived Tg, which present epitopes that differ from the plasma-derived Tg produced by thyroid tumours, and this can result in false negatives in the presence of thyroid carcinoma [196]. A second major limitation relates with the fact that 25–30% of patients with differentiated thyroid cancer have endogenous circulating anti-Tg antibodies (a three-fold greater prevalence than that found in the general population) [193], which can interfere with the assays, usually producing low or undetectable Tg levels, impairing the use of this biomarker for follow-up purposes. On the contrary, the presence of heterophile antibodies (such as human anti-mouse antibodies) may result in falsely high Tg levels [186]. Finally, total Tg serum concentration levels cannot differentiate between benign and malignant thyroid disease [197], therefore more specific biomarkers are needed for the diagnosis of thyroid cancer.

In several chronic thyroid diseases, such as cancer and autoimmunity, alterations in Tg glycosylation have been observed [64, 198]. In thyroid carcinogenesis, changes in sialylation and fucosylation, increased β1,6 branching of *N*-glycans, alterations in the content and structure of poly-LacNAc chains, and changes in *O*-GlcNAcylation have been reported (Figure 1) [63–66]. Plasma *N*-glycomes from samples of thyroid cancer, benign thyroid nodules, and matched healthy controls were analyzed by MALDI time-of-flight MS (MALDI-TOF MS) and they were found to differ between the three groups in several aspects, namely complexity, galactosylation, fucosylation and sialylation [199]. Histochemical analysis of tissue specimens from human thyroid carcinomas showed increased sialylation (especially α2,3-linked sialic acid) on follicular epithelial cells [65]. Nonetheless, Tg displayed decreased sialylation in cancer transformation (Figure 1) [67]. Furthermore, differences in the lectin binding profile between normal Tg and Tg from thyroid carcinoma have been described. Tg from thyroid carcinoma showed a lower affinity for Con A and a higher affinity for WGA, in contrast with Tg from normal thyroid tissue [200]. Asialo-glycan chains with truncated structures were also found in malignant Tg, and carbohydrate chains of higher molecular mass, which have repeating Gal-GlcNAc disaccharides and peripheral α-fucosyl residues were present in metastatic papillary carcinoma [68]. In papillary thyroid carcinoma, phosphorylated high-mannose type and hybrid-type oligosaccharides each containing one phosphate group in a diester linkage were also found to be present instead of sialylated oligosaccharides [69].

Additionally, Tg from patients with metastatic thyroid cancer showed increased binding to LCA, which has an affinity for mannose and glucose residues, allowing to discriminate between benign thyroid disease and thyroid cancer, with the LCA-binding Tg fraction being significantly lower in patients with thyroid cancer [201]. In the referred study, the immunocaptured Tg was incubated with LCA and both LCA-bound and unbound fractions were measured. The LCA-reactive Tg ratio (%) was calculated as [(total Tg concentration – LCA-nonreactive Tg concentration)/total Tg concentration × 100]. For total Tg concentration < 50 ng/mL, the LCA-reactive Tg ratio could not distinguish between healthy volunteers, patients with benign tumours, and patients with thyroid carcinoma without metastasis but, for total Tg concentration > 200 ng/mL, the calculated parameter could discriminate between benign thyroid tumours and thyroid carcinoma without metastasis or with lymph node metastasis. For total Tg concentrations between 51 ng/mL and 200 ng/mL, the LCA-reactive Tg ratio did not differ between patients with benign tumours and those with thyroid carcinoma without metastasis, but significant differences in it were observed between patients with thyroid carcinoma with lymph node metastasis and those with benign thyroid tumour or thyroid carcinoma without metastasis. Therefore, the LCA-reactive Tg ratio showed to be a valuable marker for distinguishing between thyroid carcinoma and benign thyroid tumours. The same method was applied to another set of samples, and the results confirmed that it could be used as a screening method for primary thyroid carcinoma [62]. For an LCA-reactive Tg ratio cut-off of 81.8%, with patients below this level defined as positive, the rate of positivity was 11.3% in the benign group and 61.4% in the malignant group. Total Tg serum concentration measured in the same samples did not significantly differ between benign and malignant groups, which demonstrates the enhanced performance of the LCA-reactive Tg ratio over the total Tg concentration as a sensitive and specific biomarker for thyroid cancer.

## Conclusions

The majority of approved and clinically-used biomarkers for cancer diagnosis and monitoring are serum glycoproteins that are produced and secreted by tumours. Nevertheless, the corresponding analytical assays quantify total protein concentrations, regardless of the glycans that the proteins may carry. In many disease situations, there is no clear distinction between benign and malignant status in what concerns protein concentrations, and, therefore, the assays based on the quantification of total protein concentration show insufficient sensitivity and specificity for cancer screening and early diagnosis.

As research progresses, cancer-associated glycosylation has been reported and fully characterized for many currently-used cancer biomarkers. Being a hallmark of cancer, aberrant glycosylation enables to differentiate between normal and cancer-related glycoproteins and should be exploited in order to develop more specific and accurate biomarker assays. The examples reported in this review visibly demonstrate the value of glycomic analysis for improved cancer detection and monitoring. Whenever the analytical assay combines quantification of glycoprotein serum concentration and glycoform analysis, the clinical performance significantly improves, achieving higher sensitivity and specificity percentages in discriminating benign from malignant diseases and even in differentiating malignant disease stages. This enhanced performance may further expand the use of a glycobiomarker for other clinical applications, such as screening and early diagnosis, for which it cannot be used if only the total protein concentration is measured. Moreover, the detection of multiple glycobiomarkers could achieve an even higher degree of sensitivity and specificity, as already demonstrated in several studies [202–204].

The discovery of novel cancer serum glycobiomarkers is an ongoing task, with numerous studies reporting the identification of aberrant glycoforms of glycoproteins and their potential use as clinical markers [205–207]. From all serum glycoproteins recently reported as potential cancer serum markers, haptoglobin seems to be a promising glycobiomarker, in which the strategy of combined protein quantification and glycan characterization could be employed. On one hand, increased levels of serum haptoglobin are typical of diverse solid tumours, being considered a prognostic marker [208]. On the other hand, it presents aberrant glycoforms associated with gastric [209], pancreatic [210], colon [211], HCC [212], and lung [213] cancers, which could give additional specificity and improved analytical performance to the analytical assays. Further studies are needed in order to assess this possibility.

So far, a two-step assay appears to be the method of choice for rapid and decentralized analytical tests, where the glycobiomarker is first captured by a monoclonal anti-protein antibody, providing the required specificity and allowing quantification, followed by lectin-based fractionation of the captured glycoprotein, for glycoform characterization. The first immunocapture step allows to minimize the problem of relevant cancer biomarkers being present in minute amounts in serum, masked by high-abundant proteins and glycoproteins. In the second step, lectin-based analysis of glycoforms is preferred to antibody-based analysis due to the low antigenicity of glycans. Nonetheless, some drawbacks persist, demanding further technological development. Namely, standardization of detection for uniformized clinical implementation has to be improved, together with efforts to increase automation and miniaturization of the assays.

Maturation of more sophisticated analytical techniques like MS and related methods, and their wider application in clinical analysis have enabled to perform a comprehensive characterization of cancer-associated glycans carried by glycoproteins, providing site information [214–220]. However, MS-based techniques are not yet the methods of choice for rapid and decentralized glycobiomarker quantification/characterization assays, due to the inherent instrumental and bioinformatics requirements, and high costs.

## Abbreviation

%fPSA: percentage of free prostate-specific antigen
AAL: *Aleuria aurantia* lectin
AFP: α-fetoprotein
AFP-L3%: α-fetoprotein-L3 percentage of the total α-fetoprotein
AUC: area under the curve
BPH: benign prostatic hyperplasia
CA19-9: cancer antigen 19-9
CEA: carcinoembryonic antigen
Con A: concanavalin A
CRC: colorectal cancer
CV: coefficient of variation
ELISA: enzyme-linked immunosorbent assay
GalNAc: *N*-acetylgalactosamine
HCC: hepatocellular carcinoma
LacdiNAc: *N*-acetylgalactosamine β1,4-GlcNAc
LacNAc: *N*-acetyllactosamine
LCA: *Lens culinaris* agglutinin
Le^x^: Lewis x antigen
LiBA: liquid-phase binding assay
LLOQ: lower limit of quantification
MAA: *Maackia amurensis* agglutinin
MS: mass spectrometry
MUC1: mucin 1
PSA: prostate-specific antigen
SLe^a^: sialyl Lewis a antigen
SNA: *Sambucus nigra* agglutinin
ST antigen: sialyl-Thomsen-Friedenreich antigen
STn antigen: sialyl-Thomsen nouveau antigen
T antigen: Thomsen-Friedenreich antigen
Tg: thyroglobulin
TJA: *Trichosanthes japonica* agglutinin
Tn antigen: Thomsen nouveau antigen
UEA: *Ulex europaeus* agglutinin
WFA: *Wisteria floribunda* agglutinin
μTAS: micro-total assay system

## Declarations

### Copyright

© The Author(s) 2023.
